# Adaptive Immunity and Pathogenesis of Diabetes: Insights Provided by the α4–Integrin Deficient NOD Mouse

**DOI:** 10.3390/cells9122597

**Published:** 2020-12-04

**Authors:** Salim Oulghazi, Sarah K. Wegner, Gabriele Spohn, Nina Müller, Sabine Harenkamp, Albrecht Stenzinger, Thalia Papayannopoulou, Halvard Bonig

**Affiliations:** 1 Institute for Transfusion Medicine and Immunohematology, School of Medicine, Goethe University, Sandhofstraße 1, 60528 Frankfurt, Germany or salim.oulghazi@med.uni-heidelberg.de (S.O.); sarah.k.wegner@outlook.de (S.K.W.); 2 Institute Frankfurt, German Red Cross Blood Service BaWüHe, Sandhofstraße 1, 60528 Frankfurt, Germany; g.spohn@blutspende.de (G.S.); n.mueller85@gmx.de (N.M.); s.harenkamp@blutspende.de (S.H.); 3 Institute for Pathology, University Hospital Heidelberg, Im Neuenheimer Feld 672, 69120 Heidelberg, Germany; Albrecht.Stenzinger@med.uni-heidelberg.de; 4 Department of Medicine/Division of Hematology, University of Washington, 1959 NE Pacific St., Seattle, WA 98195, USA; thalp@uw.edu

**Keywords:** VLA4, CD49d, type-1-diabetes, autoimmune diabetes, sialitis, integrin

## Abstract

Background: The spontaneously diabetic “non-obese diabetic” (NOD) mouse is a faithful model of human type-1 diabetes (T1D). Methods: Given the pivotal role of α4 integrin (CD49d) in other autoimmune diseases, we generated NOD mice with α4-deficient hematopoiesis (NOD.α4-/-) to study the role of α4 integrin in T1D. Results: NOD.α4-/- mice developed islet-specific T-cells and antibodies, albeit quantitatively less than α4+ counterparts. Nevertheless, NOD.α4-/- mice were completely and life-long protected from diabetes and insulitis. Moreover, transplantation with isogeneic α4-/- bone marrow prevented progression to T1D of pre-diabetic NOD.α4+ mice despite significant pre-existing islet cell injury. Transfer of α4+/CD3+, but not α4+/CD4+ splenocytes from diabetic to NOD.α4-/- mice induced diabetes with short latency. Despite an only modest contribution of adoptively transferred α4+/CD3+ cells to peripheral blood, pancreas-infiltrating T-cells were exclusively graft derived, i.e., α4+. Microbiota of diabetes-resistant NOD.α4-/- and pre-diabetic NOD.α4+ mice were identical. Co- housed diabetic NOD.α4+ mice showed the characteristic diabetic dysbiosis, implying causality of diabetes for dysbiosis. Incidentally, NOD.α4-/- mice were protected from autoimmune sialitis. Conclusion: α4 is a potential target for primary or secondary prevention of T1D.

## 1. Introduction

The increasing incidence and decreasing age at onset of autoimmune type-1 diabetes (T1D) [1,2] reinforces the need for a causal therapeutic intervention since euglycemia, which is always challenging to maintain with insulin replacement, is notoriously difficult to achieve in young children because of irregular life-styles, growth spurts and frequent, otherwise banal febrile infections [3,4]. When T1D is first diagnosed, significant residual islet cell mass remains [5,6] and islet cells possess relevant regenerative potential [7,8]. Interventions arresting the autoimmune process at this stage could therefore potentially still be curative [9]. Alternatively, the appearance of islet autoantibodies, sensitive harbingers of T1D development [10], in siblings of patients with T1D could trigger the initiation of islet-preserving therapies during pre-diabetes.

The adhesion molecules very late antigen-4 (VLA-4), a heterodimer of the α4 (CD49d) and the ß1 (CD29) integrins, and, to a lesser degree, LPAM-1 (α4/ß7) have been identified as exquisite targets for immune modulation in a number of autoimmune disease models [11,12,13,14], as well as in select clinical indications, namely refractory multiple sclerosis (MS) and inflammatory bowel disease (IBD) [15,16,17]. Indeed anti-functional α4 antibodies were also studied in the NOD mouse, a faithful model of human T1D. Evidence was provided that neutralizing anti-α4 rat-anti-mouse antibodies could delay, attenuate penetrance, or outright prevent T1D development [18,19,20,21]. Two basic experimental constellations were tested. Either NOD mice were transiently treated with anti-functional anti-α4 antibodies, where earlier onset of treatment and longer treatment duration seemed to affect the efficacy of the intervention [19,20,21]. Alternatively, diabetogenic NOD T-cells incubated with anti-α4 antibodies were adoptively transferred into isogeneic NOD/SCID (severe combined immunodeficiency) mice followed by further antibody treatment of the recipients. This approach similarly delayed T1D or reduced its incidence [18]. Three studies, all by the same group, addressed the role of α4 for NOD autoimmune sialitis; in their experimental set-up, function-blocking anti-α4 antibody was ineffective; the paradigm of α4 independence of NOD autoimmune sialitis is based entirely on this limited set of studies [19,22,23]. Several additional important questions could not be addressed in these models, in part due to limitations of the model per se, in part due to new technologies which have arisen since the execution of these seminal experiments. We therefore generated NOD mice deleted for α4 in the hematopoietic lineage (NOD.α4-/-) to study T1D and sialitis development and some related questions.

## 2. Materials and Methods

Mice: Diabetes-prone NOD/ShiLtJ mice (NOD) were purchased from Jackson Laboratories (Bar Harbor, ME). NOD.α4f/fTie2cre+ mice (α4-ablator mice) were generated by separately back-crossing NOD mice with the previously described C57Bl/6.α4f/f [24] and C57Bl/6.Tie2cre+ [25] mice. In the first generation, NOD males were used, to fix the Y-chromosome, in all future generations, mixed male offspring were crossed with NOD females. After 10 generations, whole genome screening for genetic purity was performed (Jackson Laboratories). Confirmed NOD.α4f/+ and NOD.cre+ mice were crossed to generate ablators. Genotyping was completed on ear punch DNA using a conventional 3-primer strategy [24]; all putatively α4-deficient mice were additionally phenotyped in blood using flow cytometry. Throughout this manuscript, we will refer to all α4-homozygous NOD mice, whether α4+/+ (irrespective of cre status), α4f/+cre- or α4f/fcre-, as “NOD” or “NOD.α4+”, to α4f/+cre+ mice as “α4 haploinsufficient”, and α4f/fcre+ as “NOD.α4-/-“. To observe spontaneous occurrence of T1D in our cohort, female NOD, haploinsufficient NOD or NOD.α4-/- mice were continuously recruited until 20 NOD.α4-/- mice had been accrued. For adoptive T-cell transfer experiments, splenocytes of diabetic NOD mice were bluntly extruded, red blood cells (RBC) lysed in hypotonic buffer, washed and immunomagnetically enriched using Miltenyi directly conjugated antibody-bead complexes, CD3-negative selection (Pan T Cell Kit II) or CD4-positive selection (CD4 microbeads) and AutoMACS according to manufacturer’s instructions (selection reagents and technology Miltenyi, Bergisch-Gladbach, Germany). Isogeneic recipients, all females aged 8–10 weeks, α4-competent or not, received 650 cGy irradiation for lymphodepletion, followed by i.v. injection of 5 × 106 CD3+ or 2.5 × 106 CD4+ splenic T-cells, as previously described [15]. Transplantation used α4-competent 10-12 week-old pre-diabetic NOD recipients who received lethal irradiation of 1050 cGy followed by i.v. transplants of 2 × 106 NOD or 10 × 106 NOD.α4-/- bone marrow (BM) cells in a volume of 200 μL, as previously reported [24]. Peritransplant, drinking water was prophylactically supplemented with 0.02% Baytril (Bayer, Leverkusen, Germany) [26]. Wherever donor cells were used, cells from 3–5 donors were pooled to reduce effects of inter-individual variability. Blood glucose level was tested weekly for spontaneous diabetes cohorts (starting week 8 for untreated and week 4 after transplantation for transplanted mice) and thrice weekly after T-cell transfer (starting 1 week after transfer) (ACCU-CHECK Aviva hardware and consumable; Roche Diabetes Care, Mannheim, Germany). Diabetes was diagnosed based on any blood glucose level >200 mg/dL on two consecutive days [15]. Prior to stool sampling, mice from the different cohorts (pre-diabetic NOD, diabetic NOD, NOD.α4-/-) were co-housed for at least one week, thereafter separated for 48 hours and from each of the mice all stool pellets were collected into sterile 1.5 mL tubes which were shock-frozen at ≤−80 °C and shipped to the Mouse Metabolic Phenotyping Center, University of California, Davis, California (MMPC). Unless otherwise noted, mice were kept in groups of up to 5 mice per cage in the vivarium of Goethe University School of Medicine under conventional (non-specific pathogen-free) conditions in filter top cages with food and water ad libitum. Mouse experiments were guided by the RRR principles, followed the Association for Assessment and Accreditation of Laboratory Animal Care International (AAALAC) guidelines for humane mouse experimentation and were approved by the animal protection authority of the state of Hessen (approval #F27/K5452).

Cellular immune system: blood was drawn from the facial vein of diabetic α4- competent NOD control mice or from 20-week-old female NOD.α4-/- mice; after RBC-lysis, peripheral blood leukocytes were stained with anti-CD45/anti-CD3/anti- CD4/anti-CD8/anti-CD44/anti-CD62L (to distinguish in the CD4+ and CD8+ compartments naïve, memory and effector cells) [27,28], as well as anti-CD335 (to detect NK-cells) [29] (all antibodies (ABs) directly fluorochrome labeled, all ABs Biolegend, Amsterdam, the Netherlands), washed and re-suspended in 7AAD-containing FACS buffer for acquisition and analysis on an LSR Fortessa flow cytometer (Becton-Dickinson). For detection of antigen-specific T- cells, lysed peripheral blood was co-stained with anti-CD45, anti-CD3 and anti-CD8 directly fluorescence-labeled antibodies (all Becton-Dickinson, Heidelberg, Germany) as well as phycoerythrin (PE)-labeled major histocompatibility (MHC)-I dextramer H-2Kd/VYLKTNVFL (from islet-specific glucose-6-phosphatase catalytic subunit-related protein, IGRP) according to manufacturer’s recommendations (Immudex, Copenhagen, DK) for acquisition/analysis on LSR Fortessa [30]. The same stainings with leukocytes from MHC-disparate C57Bl/6 mice served to establish the level of unspecific background staining (negative controls). The frequencies shown for antigen-specific CD8+ T-cells in the NOD or NOD.α4-/- mice are corrected by subtracting mean background staining from negative controls.

Humoral immune system: B-cell subtypes in BM, spleen and blood were distinguished by flow cytometry using established antibody panels (anti-CD45/anti-B220/anti-CD24/anti-CD43/ anti-IgD/anti-IgM (for detection of surface IgD/IgM)) [31,32]. To generate benchmarking data for the capacity of the α4-deficient immune system, mice received three weekly i.m. injections of 1 μg recombinant Hepatitis B surface antigen (HBs) vaccine HBVAXPRO (axicorp Pharma GmbH, Friedrichsdorf, Germany) and a booster injection in week 12, and anti-HBs titers were measured in mouse EDTA plasma 1:3 diluted in PBS after 4, 11 and 13 weeks using the ARCHITECT anti-HBs chemiluminescent microparticle immunoassay on the ARCHITECT i1000SR (Abbott, Wiesbaden, Germany) according to the manufacturer’s instructions. Anti-insulin autoantibodies were measured in mouse EDTA plasma using the IAA sandwich ELISA (Elabscience Biotechnology, Houston, Texas, USA) on the VICTOR X4 multiplate reader (Perkin-Elmer, Rodgau, Germany) as directed by the manufacturer.

Preparation of pancreas-infiltrating lymphocytes: Immediately post-mortally, the pancreas was located, the jejunum was clamped proximally and distally of the major papilla and collagenase IV buffer (1 mg/mL in 4-(2-hydroxyethyl)-1-piperazineethanesulfonic acid (HEPES)) was injected via the gall bladder. The pancreas was removed into collagenase IV buffer, digested for 15 min in a 37 °C water bath with occasional agitation, subsequently shaken to generate a homogenous cell dispersion. An excess of HEPES without collagenase was added and the suspension abruptly cooled to stop the digest, cells were washed twice, then filtered through a 70 μm filter. Under a microscope islets were picked into Roswell Park Memorial Institute (RPMI) medium with 10% fetal bovine serum and Pen/Strep and transferred to a cell culture incubator (standard conditions) to allow overnight lymphocyte migration into the medium [33]. Cells were co-stained with directly conjugated CD3 and CD49d antibody for acquisition/analysis on the LSR Fortessa flow cytometer (BD).

Histology and insulitis scoring: pancreata and submandibular glands were prepared from painlessly killed non-diabetic 8 weeks-old, newly diabetic and “chronically” diabetic (three weeks after diabetes onset) α4-competent control NOD mice as well as from 40 weeks-old NOD.α4-/- mice. They were successively paraformaldehyde-fixed (4% *v*/*v*), paraffin-embedded, sectioned into 4 μm thick slices, layered on glass slides, hematoxylin–eosin (HE)-stained and analyzed for insulitis and sialitis severity, respectively, using established morphological scores, as described [21]. Briefly, islet without lymphocytic infiltrates were scored as °0, minimal focal infiltration as °1, peri-islet infiltration of <25% as °2, peri- and intra-islet infiltration of <50% as °3, and extensive islet infiltration of ≥50%, with or without complete destruction of islet tissue and replacement by fibroblasts as °4. Sialitis was scored as number of lymphocyte infiltrates per section [34]. Scoring was blinded and performed by two independent investigators. Immunofluorescence histology used 10 μm frozen sections incubated with rabbit-anti-mouse-CD3 (OriGene, Rockville, MD, USA), rat- anti-mouse-Meca32 (BioLegend, San Diego, CA, USA) and goat-anti-rabbit Alexa488, goat-anti-rat Alexa546 and DAPI (all Invitrogen, Carlsbad, CA, USA) using standard protocols. Histological specimens were visualized on an Olympus (Tokyo, JP) BX53 microscope fitted with an Olympus SC30 camera model; image acquisition and analysis were done with cellSens software (Olympus) or with the BZ-X810 All-in- One Fluorescence Microscope, Keyence (Neu Isenburg, Germany) and imaging software BZ-X800 Analyzer. No adjustments except brightness were made.

Microbiota: frozen fecal samples were shipped on dry ice to UC Davis MMPC and Host Microbe Systems Biology Core. Total DNA was extracted using Mo-Bio (now Qiagen, Venlo, The Netherlands) PowerFecal kit. Sample libraries were prepared and analyzed by barcoded amplicon sequencing. In brief, purified DNA was PCR- amplified on the V4 region of the 16S rRNA genes using primers F319 (5′-ACTCCTACGGGAGGCAG CAGT-3′) and R806 (5′-GGACTACNVGGGTWTCTAAT-3′). High-throughput sequencing was performed with Illumina MiSeq paired end 250- pb run. The sequencing data were processed using QIIME2 for 16S based microbiota analyses (Qiime2 version 2018.6.0; QIIME2 Development Team (2017)). For quality filtering and feature (OTU) prediction, we used DADA2 [35]. Forward and reverse reads were truncated to 260 and 220 nucleotides, respectively. Representative sequences were aligned using MAFFT [36]. A phylogenetic tree of the aligned sequences was made using FastTree 2 [37]. OTUs/features were taxonomically classified using a pre-trained Naïve Bayes taxonomy classifier. The classifier was trained using the Silva 128 97% OTUs [38] for the 310F-806R region. Tables of taxonomic counts and percentage (relative frequency) were generated. Diversity analyses were run on the resulting OTU/feature biom tables to provide both phylogenetic and non-phylogenetic metrics of α and ß diversity [39] Additional data analysis (PLS-DA) and statistics were performed with R. Analyses and bioinformatics were performed by the MMPC.

**Figure 1:**
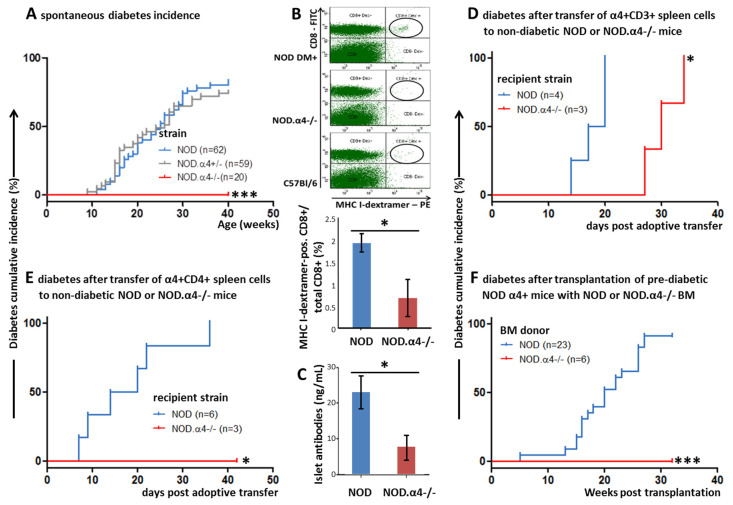
Diabetes and adaptive immune responses against islet antigens. Diabetes was diagnosed on the basis of recurrent hyperglycemia. Spontaneous diabetes development was tracked in α4-WT (NOD, blue), α4-haploinsufficient NOD (NOD.α4+/-, grey) and α4-deficient NOD (NOD.α4-/-, red) mice. The cumulative diabetes incidence after 40 weeks in the two α4-competent strains was 82% (NOD) and 75% (NOD.α4+/-), respectively (*p* = n.s.), while none of the NOD.α4-/- mice developed diabetes (*p* < 0.001 vs. both α4-competent strains) (**A**). MHC-I dextramer H-2Kd/VYLKTNVFL binding to CD8+ T-cells was assessed by flow cytometry. Shown are representative dot plots for NOD, NOD.α4-/- and MHC-disparate C57Bl/6 (negative control) mouse blood (upper panel) as well as quantitative analysis (lower panel) where dextramer-positive events in negative control blood were subtracted as background (*n* = 9 per group; *p* < 0.05) (**B**). Anti-insulin autoantibodies were quantified by ELISA (*n* = 7 per group; *p* < 0.05) (**C**). 5 × 10^6^ CD3+ splenocytes from diabetic donors were transferred into young adult non-diabetic NOD or NOD.α4-/- hosts. All recipients became diabetic, but onset of diabetes was significantly delayed in NOD.α4-/- hosts (*p* < 0.05) (**D**). 2.5 × 10^6^ CD4+ splenocytes from diabetic donors were transferred into young adult non-diabetic NOD or NOD.α4-/- hosts. NOD rapidly became diabetic, while NOD.α4-/- were immune (*p* < 0.05) (**E**). Lethally irradiated pre-diabetic young female NOD mice received bone marrow transplants from NOD or NOD.α4-/- donors. Recipients of NOD bone marrow became diabetic with typical kinetics, while recipients of NOD.α4-/- bone marrow were protected (*p* < 0.001) (**F**). Asterisks indicate statistical significance at the 0.05 (*) or 0.005 (***) level.

Statistics: descriptive statistics and Student’s *t*-tests, with Bonferroni correction for multiple testing where applicable, were calculated in Excel (Microsoft, Redmond, WA). Non-parametric values were compared using Mann–Whitney’s *U*-test calculated with the online tool on www.socscistatistics.com. Kaplan–Meyer statistics were calculated using the log–rank (Mantel–Cox) test in Prism (GraphPad Software, San Diego, CA). With the exception of the experiments shown in Figure 1D and Figure S4, all cohort studies were performed in at least two independent experiments; the cumulative number of animals from all experiments is given either directly in the figure panel or the accompanying legend. A *p* < 0.05 was considered statistically significant.

## 3. Results

### 3.1. Generation of NOD.α4-/- Mice

Hematopoietic specific ablation of the α4 integrin under the control of the tie2 promoter was described previously [24]. Tie2Cre+ and α4f/f mice were individually inbred into the NOD background for 10 generations. NOD strain background was confirmed using whole genome screening (Figure S1A); the strains we then crossed to generate NOD.α4f/fTie2cre+ (NOD.α4-/-) mice (Figure S1B,C for genotyping strategy and exemplary results). NOD.α4-/- mice were efficiently α4 ablated in the hematopoietic lineage, specifically also on T-cells (Figure S1D). Albeit being born at slightly less than Mendelian ratios, NOD.α4-/- were normal-sized and healthy-appearing.

The α4-/- cellular immune system was previously tested and its overall functionality confirmed for C57Bl/6 mice, although several models of autoimmune disease were markedly attenuated in α4-/- mice [40,41]. We assessed distribution of T-cells in NOD.α4-/- blood and spleen of naïve, effector and memory cells in both cytotoxic and helper cell compartments, as well as relative frequency of T-cells (Figure S2A–C) and found them to be very similar in NOD and NOD.α4-/- cells. Both strains also had similar numbers of NK cells (Figure S2D). The α4-/- humoral immune system has received less attention; for NOD.α4-/- mice, here we show quantitatively normal B-cell maturation in spleen, blood and bone marrow (BM) (Figure S3A–C, respectively) as well as demonstrating—in an innovative murine immunization model—that α4-deficiency does not impair the humoral immune response including B-memory function per se (Figure S3D), although antibody titers were significantly lower in NOD.α4-/- than in NOD mice (*p* < 0.05).

### 3.2. NOD.α4-/- Mice Are Protected from Autoimmune Diabetes

Twenty female NOD.α4-/- mice and all concurrent female NOD (wild-type; *n* = 62) or NOD.α4+/- (α4 haplo-insufficent; *n* = 59) mice were continuously accrued and observed for a total of 40 weeks for development of T1D. NOD and NOD.α4+/- became diabetic with indistinguishable kinetics (*p* = n.s.), with the first mice manifesting at 9–10 weeks of age, reaching peak cumulative incidence of 75–80% at age 40 weeks and dying or requiring painless killing within 1–3 weeks of diabetes onset. By contrast, NOD.α4-/- mice were completely protected from diabetes (cumulative incidence, 0%; *p* < 0.001 (lLog–rank test) vs. NOD and NOD.α4+/-; Figure 1A). Because of the absence of a distinctive (e.g., attenuated) phenotype of the α4 haplo-insufficient mice, all further experiments considered haplo-insufficient mice as α4-competent. Henceforth, we will solely distinguish between NOD (α4-competent) and NOD.α4-/- mice.

### 3.3. Adaptive Cellular and Humoral Immune Responses of NOD.α4-/- Mice against Islet Cell Antigens

**Figure 2:**
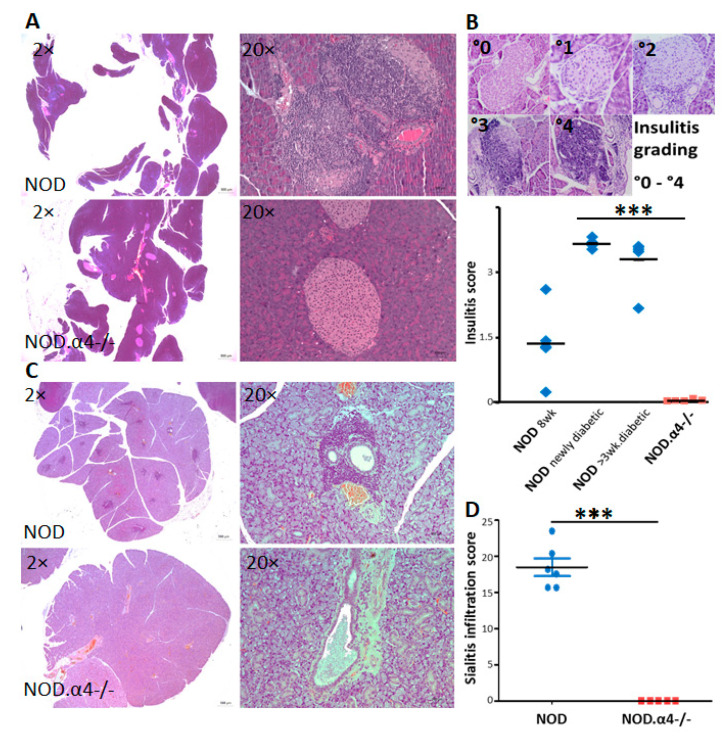
Histological analysis of pancreas islets and submandibular glands. Histological evaluation of lymphocyte infiltration of pancreas islets (**A**,**B**) and submandibular glands (**C**,**D**). Exemplary appearance of diabetic NOD or age-matched NOD.α4-/- pancreas (**A**) and quantitative analysis of insulitis severity in the indicated groups (**B**). Each dot represents the average insulitis score from multiple non-consecutive sections from one animal, the bar marking the mean (n = 5 (NOD) to 8 (NOD.α4-/-) animals per group; *p* < 0.005 for NOD.α4-/- vs. each of the NOD groups, *p* = n.s. between NOD groups). Inset images of islets in (**B**) exemplify the 5-grade histological scoring system between 0 and 4. Exemplary appearance of NOD or NOD.α4-/- submandibular glands (**C**) and enumeration of sialitis score (**D**). Each dot represents the average sialitis score from multiple non-consecutive sections from one animal, the bar marking the mean (*n* = 5–6 animals per group; *p* < 0.001). Asterisks indicate statistical significance at the 0.005 (***) level.

**Figure 3:**
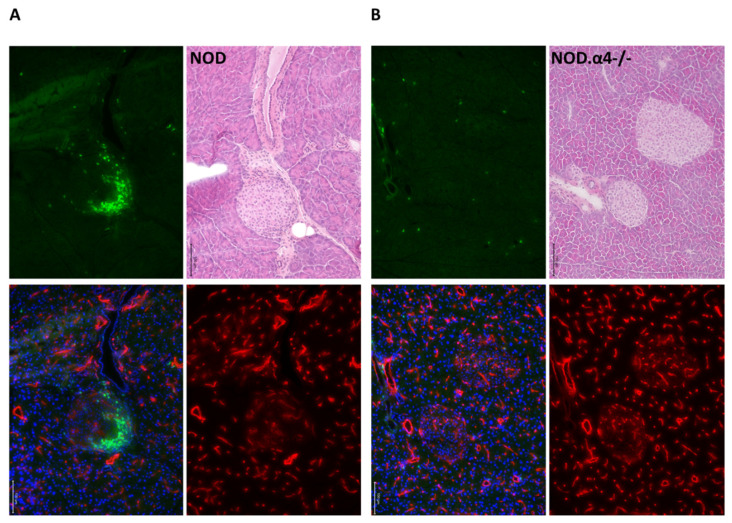
Pancreas immunofluorescence histology of young adult non-diabetic mice. Pancreas from NOD (**A**) and NOD.α4-/- (**B**) young adults is shown, with a prominent lymphocyte infiltrate in (**A**). Adjacent sections were used for hematoxylin–eosin (HE) or immunohistochemistry staining (CD3: green, Meca32: red, merged fluorescence image including DAPI; 20× original magnification; 100 μm size bars in image).

**Figure 4:**
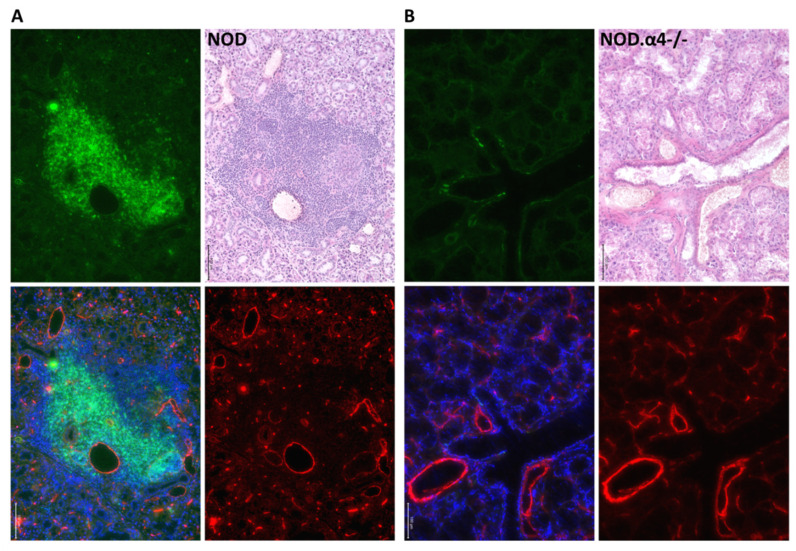
Submandibular gland immunofluorescence histology of young adult non-diabetic mice. Submandibular gland from NOD (**A**) and NOD.α4-/- (**B**) young adults is shown, with a prominent lymphocyte infiltrate in (**A**). Adjacent sections were used for HE or immunohistochemistry staining (CD3: green, Meca32: red, merged fluorescence image including DAPI; 20× original magnification; 100 μm size bars in image).

In spite of their diabetes resistance, NOD.α4-/- mice tested positive for both circulating islet-antigen specific cytotoxic T-cells (Figure 1B) and islet autoantibodies (Figure 1C), albeit both quantitatively significantly less than the α4-competent counterparts (*p* < 0.05 for both). However, these islet-antigen specific α4-/- T-cells were apparently incapable of infiltrating islets, while α4 competent NOD mice showed significant insulitis already at the age of 8 weeks of life (Figure 2A,B and Figure 3A,B; NOD.α4-/- vs. pre-diabetic 8 week-old NOD (*p* = 0.0037), vs. newly diabetic (*p* = 0.0006) and vs. longer-term diabetic (*p* = 0.0007) NOD mice; comparison of the three NOD cohorts with one another: *p* = 0.097–0.47, n.s.). NOD.α4-/- mice were also protected from the characteristic autoimmune sialitis which in NOD mice was characterized by large T-lymphocyte infiltrates at young adult age and eventually progressed to virtually complete destruction of the acini (Figure 2C,D and Figure 4A,B; *p* < 0.001).

### 3.4. Adoptive Transfer of a Mix of Diabetogenic α4+ Helper and Cytotoxic T-Cells, but Not α4+ Helper T-Cells Alone, Induce Diabetes in NOD.α4-/- Mice

Our knock-out mice provided the opportunity to perform some unique adoptive immune transfer experiments that could not be addressed with anti-functional antibodies. Using NOD or NOD.α4-/- mice as recipients of negatively selected CD3+ splenocytes from diabetic mice, both recipient groups developed diabetes within two (NOD) or four (NOD.α4-/-) weeks. The delay was statistically significant (Figure 1D; *p* < 0.05). When the diabetic NOD.α4-/- hosts were bled, their pancreata were harvested, and host vs. donor T-cells were analyzed, T-cells isolated from islets were exclusively donor-derived, i.e., α4+, despite representing only a minor fraction (13.6%) of T cells in the blood (Figure S4). We next tested the ability of α4+/CD4+ spleen T-cells to cause diabetes, with a most remarkable outcome: all NOD hosts developed diabetes, as before with very short latency, while NOD.α4-/- recipients were completely protected (Figure 1E; *p* < 0.01).

### 3.5. α4 Ablation Protects Pre-Diabetic NOD Mice from Diabetes

As α4 blockade is efficacious in established autoimmune disease like multiple sclerosis and inflammatory bowel disease in humans, we furthermore asked whether α4 ablation could also prevent autoimmune diabetes in young adult pre-diabetic NOD mice, i.e., in mice with established insulitis but with residual islet cell function and thus still normoglycemic. Eight to ten week-old female NOD hosts were radio-conditioned followed by transplantation with young female α4 competent non-diabetic NOD or NOD.α4-/- bone marrow. Both engrafted promptly and completely, and recipients of α4 competent NOD bone marrow developed diabetes with similar kinetics and cumulative incidence as for spontaneous NOD diabetes. Thus, in agreement with published work [42], lethal irradiation and transplantation by itself does not affect the outcomes as most NOD bone marrow recipients became diabetic 12–25 weeks after transplantation. All NOD mice reconstituted with α4-/- hematopoiesis, in contrast, remained diabetes-free (Figure 1F; *p* < 0.001).

### 3.6. Pre-Diabetic NOD Mice Have Normal Microbiota

**Figure 5:**
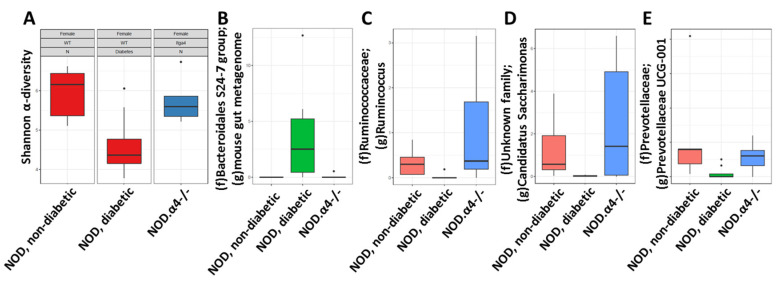
Microbiota analysis. Feces from 5 non-diabetic NOD, 5 NOD.α4-/- and 9 diabetic NOD mice were subjected to microbiota analyses. Microbiota diversity of diabetic mice was restricted (Shannon α-diversity *p* < 0.05 diabetic NOD compared to both non-diabetic groups; *p* = n.s. between non-diabetic NOD and NOD.α4-/-) (**A**) and marked by appearance (**B**), extinction (**C**,**D**) or quantitative depletion (**E**) of unique taxa (ANCOM score ≥ 0.7 for all four; box plot marking interquartile range and median; Dots mark outlier values).

Altered microbiota have been described in several autoimmune disorders including diabetes. Whether the autoimmune disease is a cause or consequence of the dysbiosis remains unclear [43,44,45]. Our model afforded us the unique opportunity to co-house α4-competent and -deficient mice. Over time, some of the α4-competent ones eventually became diabetic. Mice exhibiting coprophagia, co-housing were expected to result in maximally similar microbiota. Indeed non-diabetic (or more precisely, pre-diabetic) NOD and diabetes-resistant NOD.α4-/- mice had essentially indistinguishable microbiota. Similarity extended to both α-diversity (Shannon α-diversity; *p* = 0.75) and abundance or absence of certain components. By contrast, α-diversity was markedly restricted in diabetic NOD mice (*p* = 0.02 and 0.04 compared to non-diabetic NOD and NOD.α4-/- mice, respectively, Figure 5A). Moreover, certain genera were completely extinct, such as Ruminococcus, Prevotella and Candidatus Saccharimonas, or over-represented (Bacteroidales S24-7 group) in the feces of diabetic NOD mice (Analysis of Composition of Microbiomes (ANCOM) scores 0.7 or greater for all; Figure 5B–E).

## 4. Discussion

Several studies reported effects of anti-functional anti-integrin antibodies in mice which experiments with genetic models subsequently unraveled as artifacts (for a recent example, see: [46]). Therefore, although work with anti- functional antibodies in NOD mice [15,19,20,21] had in principle indicated contributions of α4 integrin to insulitis and diabetes (although their magnitude was discussed controversially), we sought to reappraise the issue with an NOD.α4 knock-out mouse. Furthermore, the knock-out mouse could address certain questions which were inaccessible with anti-functional antibodies or had previously remained unanswered. Specifically, hematopoietic integrin knock-out mice allow the separation of effects of circulating vs. resident and hematopoietic vs. stromal cells, as well as interrogation of contributions of isolated sub-populations. The unique, or novel, insight provided by the newly generated NOD.α4-/- mouse is discussed below.

For one, certain short-comings of published studies with xenogeneic anti-functional antibodies in NOD mice [15,19,20,21] could not be ruled out by the design of the published studies, including immunological reactions which could cause anti-diabetic inflammatory responses or opsonization and lymphodepletion, or simple pharmacological effects such as dosing and dose interval. In the published work, protection was mostly only relative and highly variable between studies; the n in most studies was small [15,21]. As we are conclusively showing in a large cohort, α4 ablation affords perfect protection from T1D and autoimmune insulitis, thus, definitively validating prior work with our distinct experimental approach (Figure 1A and Figure 2A,B).

Secondly, some tools for more mechanistic assessment of anti-islet immune responses were until quite recently not available so that earlier work regarding α4 in T1D had predominantly provided phenotypic analyses [15,19,20,21]. Using MHC multimer reagents (H-2Kd with peptide VYLKTNVFL from IGRP), we clearly identified islet antigen-specific T-cells in NOD.α4-/- mice. To study the NOD.α4-/- humoral immune system, we developed a novel, highly sensitive, specific and quantitative immunization model. The assay informs of the principle ability of the mice to initiate adaptive humoral responses and to establish humoral memory. Indeed we know that patients treated with anti-functional anti-α4 antibodies are capable of B-cell responses [47]. The attenuated humoral response to the immunization observed here (Figure S3D) is proportionally reflected by the reduced islet autoantibody titers (Figure 1C). Thus, α4 deficiency prevents neither cellular nor humoral responses against islet autoantigens. Instead, α4-/- T-cells fail to migrate into the target tissue (Figure 2A,B and Figure S4). The observation teaches that adaptive immunity against islet antigens arises in extra-pancreatic compartments and is not critically dependent on α4 integrin. Moreover, islet autoantibodies by themselves do not induce diabetes. This conclusion is in line with clinical observations in patients with established MS and IBD, where therapeutic α4-blockade remains effective in the presence of significant numbers of antigen-specific T-cells in and inflammation of target tissues [16,17,18,47]. With our transplant experiments, whereby pre-diabetic α4+ recipients were made diabetes-resistant with transplants from NOD.α4-/- donors, we extend this paradigm to T1D (Figure 1F).

Thirdly, a genetic model can target integrins on selected immune cell species as opposed to the invariably ubiquitous inhibition achieved by antibodies. We thus performed some unique experiments with mixed chimeras for α4+ and α4-/- T-cells. Transfer of α4+ splenocytes from diabetic donors to NOD or NOD.α4-/- mice induced diabetes with 100% penetrance, albeit with a marked delay in the NOD.α4-/- mice (Figure 1D). We take this temporal delay to indicate differential recruitment velocity of autoaggressive T-cells to inflamed (in the pre-diabetic α4+ recipients) vs. bland (in the α4-/- recipients) islets. Alternative or complimentary explanations for the differential kinetics of adoptively transferred diabetes include overall more rapid activation and proliferation of these T-cells in the inflammatory milieu of the pre-diabetic NOD mouse although one might expect the radiation-induced inflammation to override subtle systemic effects of insulitis. Possibly, reduced islet cell reserve in pre-diabetic hosts can contribute to relative disease acceleration, less optimal interaction with contributing α4-/- than with WT non-T-host cells to slight attenuation. Importantly, although markedly under-represented in blood, all T-cells recovered from pancreata of these mixed chimeras were α4+ (Figure S4). Thus, even inflamed islets infiltrated with α4+ T-cells cannot co-recruit α4-/- T-cells.

While transfer of diabetic donor CD8+ cells alone cannot elicit diabetes [48], a highly variable diabetogenic potential of diabetic donor CD4+ cells has been reported [48,49,50,51]. In some cases, co-injected contaminating CD8+ T-cells could be imputed [49]. Importantly, these data were generated with immunodeficient NOD/SCID hosts which lack endogenous lymphocytes which the donor cells could coopt and whose antigen presentation is defective [52]. From these data, Janeway et al. conclude that although in principle CD4+ T-cells are capable of T1D induction, this process is much more efficient in the presence of CD8+ T cells [48]. In our immunocompetent NOD hosts, transfer of highly purified CD4+ cells from diabetic donors caused diabetes in all α4+, yet none of the α4-/- recipients. Thus CD4+ T-cells are actually quite efficient at inducing diabetes if they can recruit and instruct additional α4+ host immune cells, presumably CD8+ lymphocytes. α4-/- immune cells, whether innate or adaptive, cannot support α4+/CD4+ to induce islet cell destruction.

Fourth, the reciprocal relationship between host and gut microbiota has been the focus of a significant body of recent work [43,44,45]. The association of a given disease with recurrent alterations of the microbiota has sometimes been interpreted as indicative of a causal relationship: According to that hypothesis, the aberrant microbiota was hypothesized to arise spontaneously and to exacerbate the pathology [53,54]. Clearly, dysbiosis with pathogenic bacteria can attenuate diabetes proneness in the NOD mouse [55]. Our NOD.α4-/- mice afforded the opportunity to study the microbiota in unperturbed co-housed pre-diabetic, newly diabetic or diabetes- resistant NOD mice. We pursued two aims, the first seeking to ascertain whether a universal “diabetic microbiota” exists, by comparing our data with published work in other diabetes models [43,44,45,53,54,55,56]. Secondly, our genetic model should be able to answer the question of cause and causality of diabetes and dysbiosis. Indeed, we observed a marked dysbiosis in the diabetic NOD mice, with significantly reduced diversity and selection/de-selection of certain bacterial genera (Figure 3). Both lack of diversity and the specific changes in the microbiota are similar in nature and magnitude, and they also affect some of the same genera as were previously reported for other diabetes models [43,44,45,53,54,55,56]. Common denominators include the appearance of Bacteroidales and extinction of Prevotella in diabetic NOD mice [53,54,56]. Pre-diabetic and diabetes-resistant NOD mice’s microbiota, however, was indistinguishable. Thus the “diabetic” flora only establishes itself once the mice are hyperglycemic. The scientific community is currently beginning to understand nutrient preference of microbial taxa in situ; apparently certain gut metabolites select or de-select specific microbes. Thus, Cand. Saccharimonas, extinguished from the microbiota of the diabetic hosts, [57] negatively correlates with methylamine in stool, with methylamine being a product of amine catabolism which is increased in diabetes. The aggregate evidence thus suggests that in the model presented here, abnormal microbiota is consequence, not a cause of diabetes in NOD mice, likely secondary to an altered fecal metabolome.

Fifth, our model conclusively demonstrates for the first time the critical dependence on α4 integrin for development of autoimmune sialitis in NOD mice. In earlier work [19,20], administration of α4-blocking antibody for the first four weeks of life had failed to affect frequency and severity of sialitis at 42 weeks of age. Since the same antibody regimen had provided long-term protection from diabetes, the authors had thus concluded about α4-independence of NOD sialitis. The probable explanation for these divergent findings is that likely long-term α4-blockade is required to prevent sialitis.

Sixth, protection of pre-diabetic recipients of α4-negative hematopoietic transplants documents the potential of α4-targeted therapy to prevent diabetes even after sensitization and islet cell infiltration have occurred, i.e., in a clinically relevant scenario. Similar experiments in new-onset diabetic mice failed due to the rapid disease progression after diagnosis.

Short-comings of the NOD model as a model of human T1D deserve brief mentioning. The strain is characterized by numerous immunological defects, including aberrant down-regulation of MHC in response to IFNγ [58], apoptosis-deficient lymphocytes [59], abnormal function of antigen-presenting cells and natural killer-cell dysfunction [60], as well as complement C5 deficiency [61], together translating into a general defect in the development of regular immune tolerance [62]. Possibly in part due to these abnormalities, but also due to a timing which is hard to reproduce in humans, i.e., long before diabetes onset [9], of the long list of interventions capable of attenuating diabetes risk in NOD mice most have failed to be confirmed in humans [9]. With respect to α4 we believe, however, that the ability of α4-blocking antibody to modify the course of certain established autoimmune diseases predicts that the situation may be different. We thus predict that α4 blockade during pre-diabetes (after islet-specific autoantibodies can be detected) or even in recent-onset diabetes can be arrested or reversed. Alternative animal models of spontaneous diabetes, most famously the biobreeder (BB) rat [63], have been described, each with their own idiosyncrasies, but for the work presented here the NOD model appears to be meaningfully informative.

## 5. Conclusions

In summary, we show that NOD.α4-/- mice are diabetes resistant despite developing adaptive immunity, albeit attenuated, against islet cell autoantigens. Transfer of diabetogenic CD4+ T-cells did not cause diabetes in NOD.α4-/- mice. Diabetic NOD mice have a “diabetic” microbiota which is markedly distinct from the microbiota of co-housed pre-diabetic NOD and NOD.α4-/- mice, implying that in the absence of external selection pressure on the microbiome (e.g., with antibiotics), host factors—likely nutritional—select the microbiota. NOD.α4-/- mice are protected against autoimmune sialitis. The data provided in this paper have immediate translational potential, since relatively safe, tolerable and highly effective α4-targeting reagents of pharmaceutical quality, i.e., the anti-functional anti-α4-antibody Natalizumab, are readily available. Certainly during the pre-diabetic phase characterized by normoglycemia with circulating islet antigen antibodies, α4-blockade would be expected to provide secondary diabetes prevention. We also posit that therapeutic α4-blockade in children with recent-onset diabetes will be able to preserve the residual islet cell mass and allow for its regeneration, thus at least delaying T1D until a less fragile age, ideally beyond puberty.

## Supplementary Materials

The following are available online at https://www.mdpi.com/2073-4409/9/12/2597/s1, Figure S1: Generation of NOD.α4-/- mice, Figure S2: T- and NK-cells in NOD and NOD.α4-/- mice, Figure S3: B-cell maturation and function, Figure S4: Adoptive transfer of CD3+ T-cells from diabetic NOD donors causes insulitis but fails to recruit α4-/- T-cells to islets.
