# Supplementary material for: Adaptive Immunity and Pathogenesis of Diabetes: Insights Provided by the α4–Integrin Deficient NOD Mouse
Source: Cells. 2020 Dec 4;9(12):2597. doi: 10.3390/cells9122597 (PMC7761835; doi:10.3390/cells9122597)
Supplement: Supplementary file 1 [file cells-09-02597-s001.pdf]

cells MDPI

Supplemental Figures

Figure S1

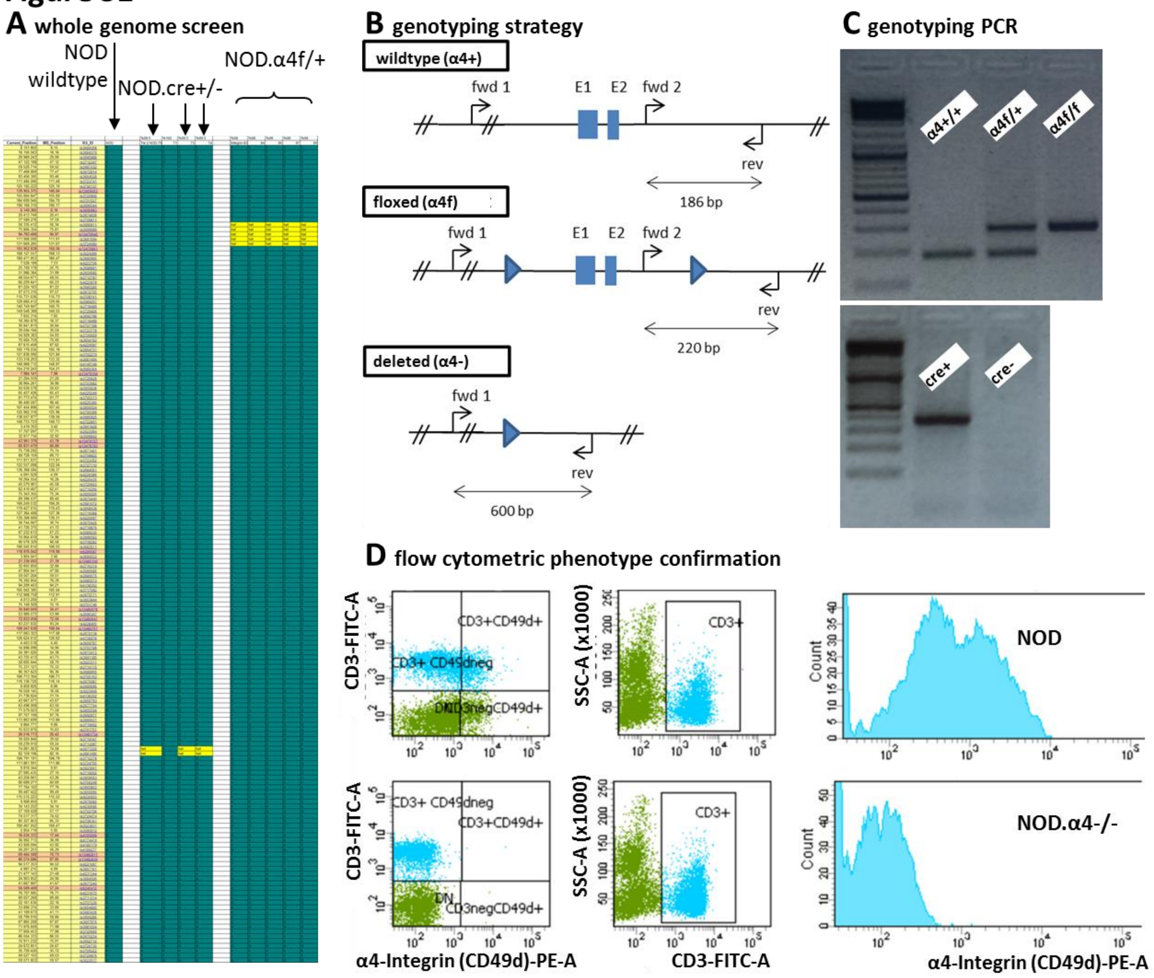

Figure S1. Generation of NOD.α4-/- mice

The previously described α4f/f and Tie2cre mice were separately bred to the NOD background. Pure NOD background except for a single copy of Tie2cre or the α4f allele was confirmed by whole-genome screen (**A**); mice were then crossed to generate ablators. The genotyping strategy, allowing distinction of the WT, targeted (f) and deleted (-) alleles, is shown in (**B**), typical genotyping results in (**C**). α4-deletion in the hematopoietic lineage in mice genotyped as α4f/fcre+ was confirmed by flow cytometry of peripheral blood leukocytes. Shown are representative results for a NOD.α4+ and a NOD.α4-/- (**D**).

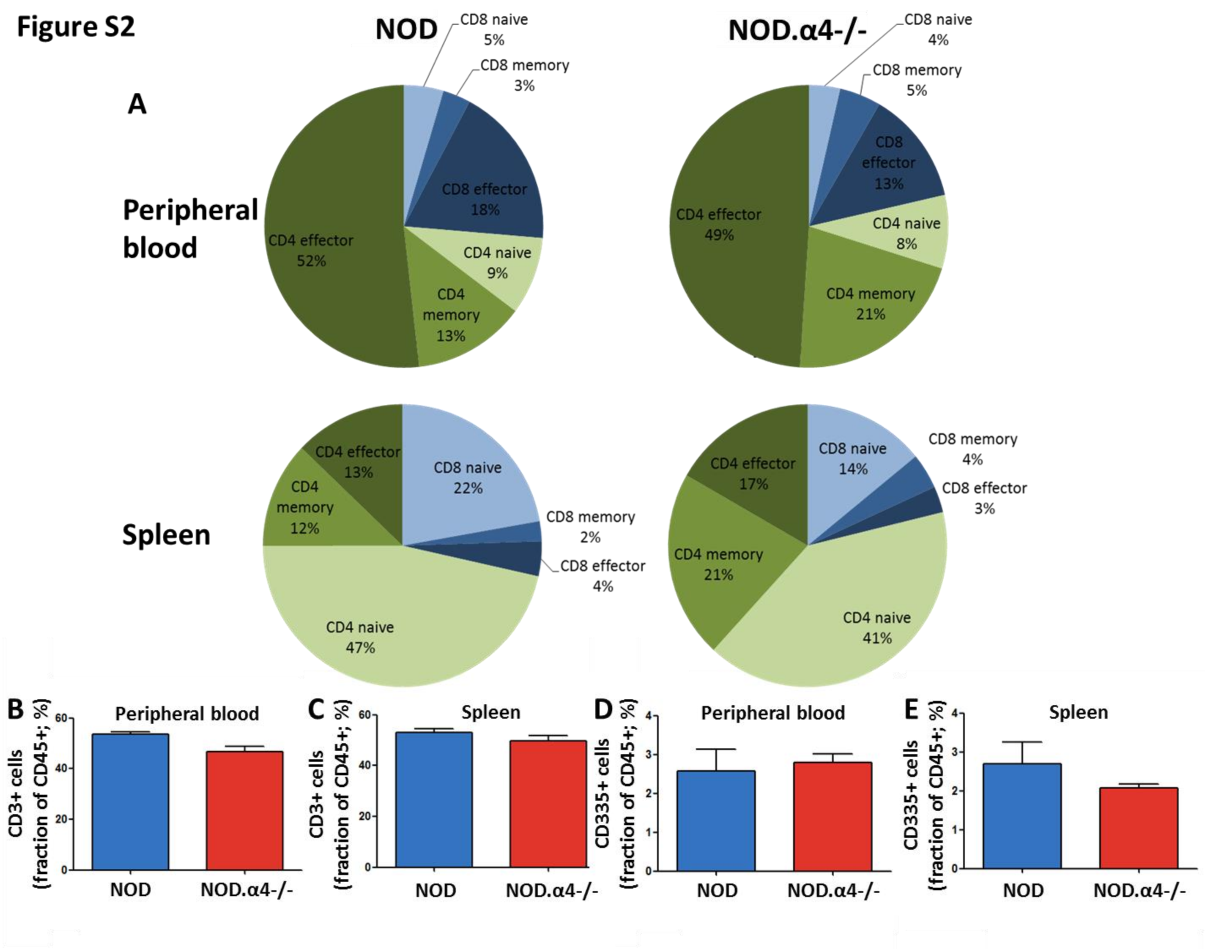

Figure S2. T- and NK-cells in NOD and NOD.α4-/- mice

Phenotypic T-cell subtype distribution was grossly similar in blood and spleen of NOD and NOD.α4-/- mice (**A**), as were total T-cell (**B, C**) and NK-cell (**D, E**) frequencies in blood (**B, D**) and spleen (**C, E**) (mean (**A**) or mean±SEM (**B-E**); n=4-5 per group). In (**A**), blue and green represent CD4+ and CD8+ T-cells, respectively, light to dark shading depicts naive, memory and effector cells, respectively.

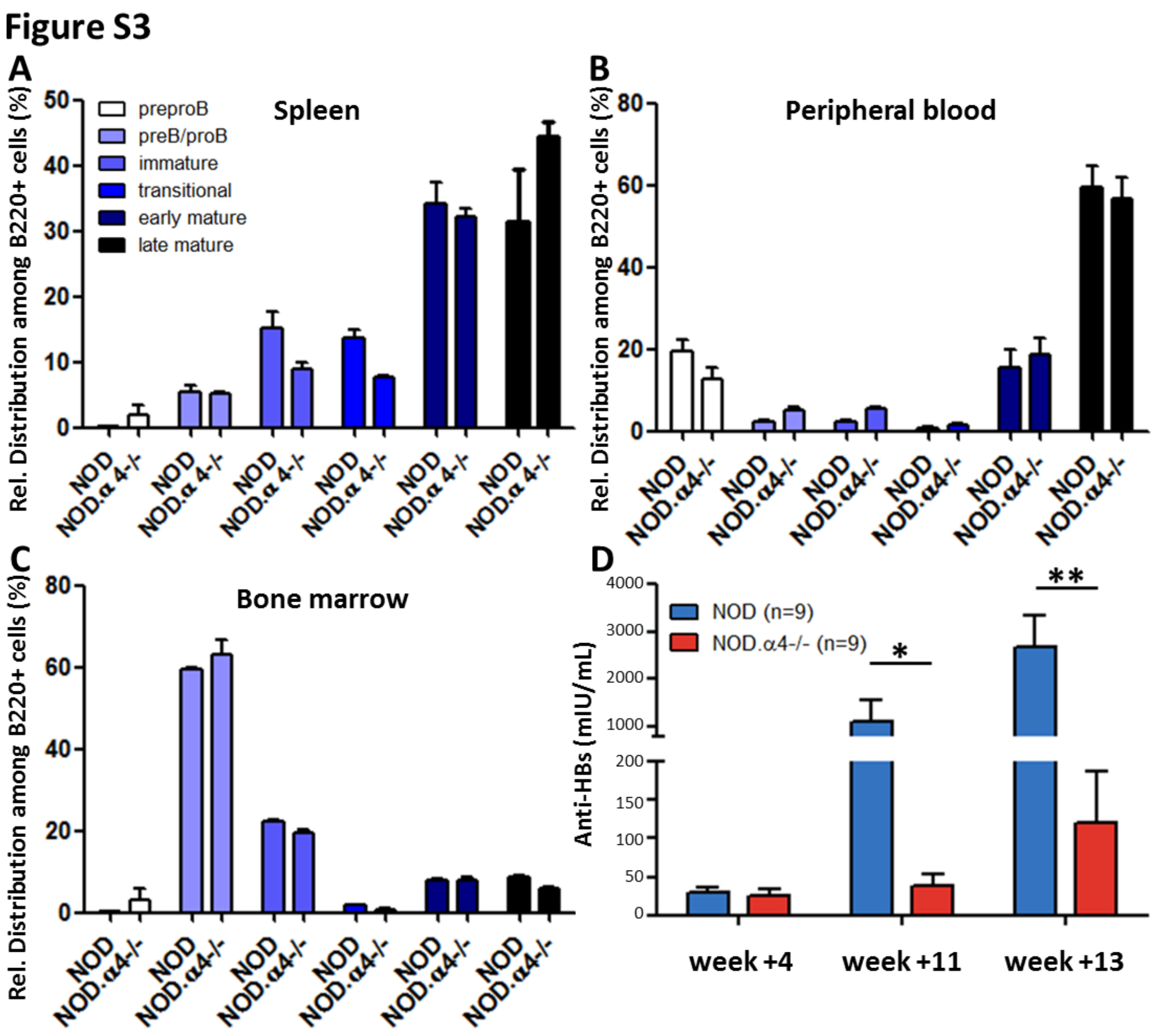

Figure S3. B-cell maturation and function

B-cells in spleen (**A**), blood (**B**) and bone marrow (**C**), were analyzed phenotypically; no significant differences were observed (mean±SEM; n=4-5 per group). Immunization of NOD or NOD.α4-/- mice with rHBs vaccine (weekly weeks 0-3, boost week 12) induced immediate and long-term humoral responses, as well as they were boostable, although quantitatively NOD.α4-/- mice responded much less strongly at the later time points (**D**) (mean±SEM; n=9 per group; $p<0.05$ at 11 weeks, $p<0.01$ at 13 weeks).

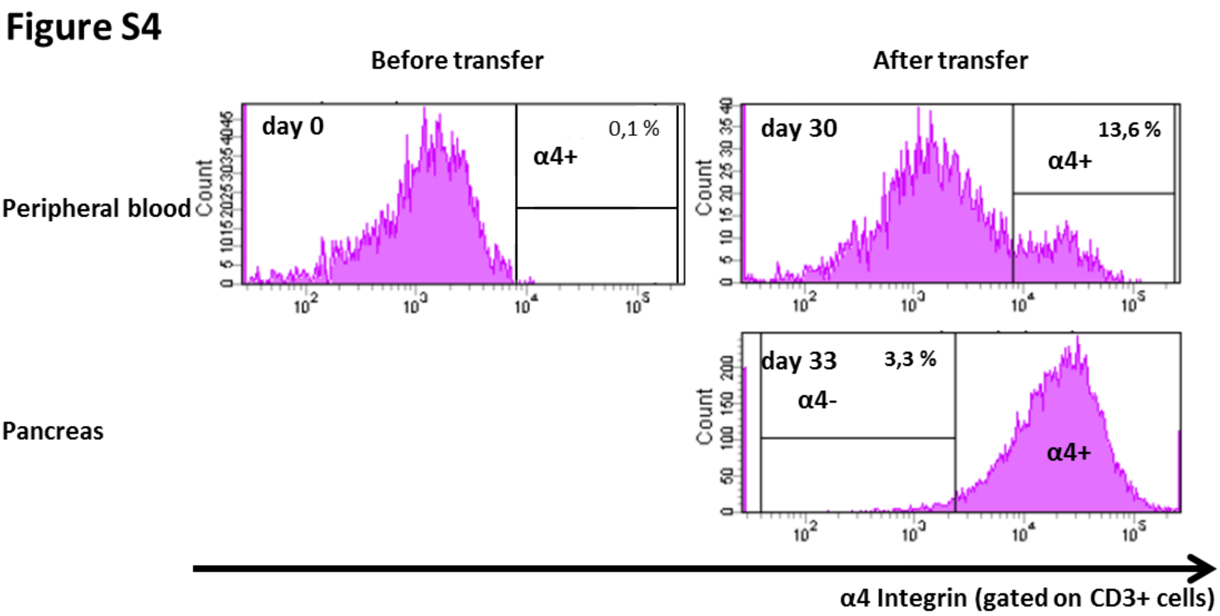

Figure S4. Adoptive transfer of CD3+ T-cells from diabetic NOD donors causes insulitis but fails to recruit ***α*****4-/- T-cells to islets**

Transfer of CD3+ splenocytes from diabetic NOD mice into NOD.*α*4-/- repopulates only 13.6% of the peripheral blood lymphocyte compartment (upper row) while CD3+ cells recovered from pancreas islets are exclusively donor-derived (bottom row). Pooled cells from islets from three animals (one experiment).

© 2020 by the authors. Submitted for possible open access publication under the terms and conditions of the Creative Commons Attribution (CC BY) license (http://creativecommons.org/licenses/by/4.0/).
